# Clinicopathological association of CD93 expression in gastric adenocarcinoma

**DOI:** 10.1007/s00432-024-05874-4

**Published:** 2024-08-27

**Authors:** Yun Shen, Yahui Wu, Mengfei Hao, Minghan Fu, Kai Zhu, Panru Luo, Jinsheng Wang

**Affiliations:** 1 Department of Pathology, People’s Hospital of Tongling City, Tongling, Anhui China; 2 Department of Pathology, Heping Hospital Affiliated to Changzhi Medical College, Changzhi, Shanxi China; 3 Department of Pathology, the First Clinical College of Changzhi Medical College, No.161 Jiefang East Street, Changzhi, Shanxi China; 4 Key Laboratory of Esophageal Cancer Basic Research and Clinical Transformation, Shanxi Provincial Health Commission, Changzhi, Shanxi China; 5 Department of Pathology, Yueyang Central Hospital, Yueyang, Hunan China

**Keywords:** Gastric adenocarcinoma, CD93, Immunohistochemistry, Prognosis, Angiogenesis

## Abstract

**Aims:**

CD93 was recently identified as a promising therapeutic target for angiogenesis blockade in various tumors. Herein, we aimed to investigate the expression and clinicopathological significance of CD93 in gastric adenocarcinoma.

**Methods:**

The gene expression of CD93 gastric adenocarcinoma was assessed using The Cancer Genome Atlas (TCGA) dataset. We then analyzed CD93 expression in 404 cases of gastric adenocarcinoma using immunohistochemistry. Clinicopathological associations and prognostic implications of CD93 expression were further investigated.

**Results:**

Using the TCGA dataset, we observed a significantly elevated CD93 gene expression in gastric adenocarcinoma compared to normal gastric tissues. The immunohistochemistry assay revealed a highly variable CD93 expression among patients with gastric adenocarcinoma, consistently demonstrating higher intratumor expression than in adjacent normal tissues. Notably, CD93 was predominantly expressed on the membrane of CD31^+^ vascular endothelial cells. Furthermore, patients with higher CD93 expression demonstrated significantly poorer overall survival. Accordingly, higher CD93 expression was associated with deeper invasion and a higher possibility of lymph node metastasis and developing tumor thrombus. Cox proportional hazards regression suggested CD93 expression was an independent predictor for the prognosis of patients with gastric adenocarcinoma.

**Conclusions:**

Our study revealed a significantly higher CD93 expression in gastric adenocarcinoma when compared with adjacent normal gastric tissues, and demonstrated its predominant expression on vascular endothelial cells. Our findings also highlighted the clinicopathological significance of CD93 in gastric adenocarcinoma, shedding light on a potential therapeutic target.

## Introduction

Gastric cancer (GC) is the global leading cause of cancer-related mortality, resulting in an estimated 769,000 deaths per year, with gastric adenocarcinoma representing the main pathological entity (Sung et al. 2021). Although chemotherapy has shown remarkable success in eliminating progression and preventing recurrence, the clinical benefit of chemotherapy for advanced GC remains compromised. Furthermore, chemotherapy commonly leads to some adverse events, such as serious neutropenia and liver dysfunction, leading to the intolerance of chemotherapy. Recently, there has been an increasing recognition that angiogenesis significantly influences tumor progression and the response to anti-tumor therapies since pathological angiogenesis contributes persistent nutrients and oxygen for tumor (Ramjiawan et al. 2017). In this regard, angiogenesis blockade can inhibit the growth of capillary network and restrict tumor invasion. Although the combination of angiogenesis blockade and conventional chemotherapy has demonstrated significant improvement in overall response rate and progression-free survival, angiogenesis blockade appears to lead to some adverse effects for patients with GC, including hypertension, gastrointestinal hemorrhage, epistaxis, and proteinuria (Shan et al. 2016). More importantly, the benefits of angiogenesis blockade based on current targets, especially vascular endothelial growth factor (VEGF), are limited in patients with advanced GC. Therefore, there is an urgent need to identify novel targets for inhibiting neovascularization of GC.

CD93 is a member of the C-type lectin transmembrane protein family and is expressed on the membrane of multiple cell lineages, especially endothelial cells of tumor vessels. Prior studies have revealed that CD93 was mainly expressed on the surfaces of endothelial cells and several immune cell populations, including monocytes, neutrophils, and microglia. In addition, a recent study indicated that CD93 was also expressed on the cytotrophoblast and guided it migration in an integrin-dependent manner (Fantone et al. 2022). More importantly, the overexpression of CD93 on tumor-associated blood vessels has been proven to be associated with advance tumor stage and poor prognosis in multiple cancer types, including lung squamous cell carcinoma (Tossetta et al. , 2023; Qu et al. 2024). Furthermore, elevated expression of CD93 has been observed in multiple non-cancerous diseases, including cardiovascular disease and stroke (Piani et al. 2023). A very recent study has identified the recruitment of a cluster of smoking-associated CD93 positive monocytes in lung upon tobacco exposure (Corleis et al. 2023). Interestingly, since membrane CD93 could be cleaved into a soluble form (sCD93), it has been used as a biomarker for both cancerous and non-cancerous diseases. For instance, serum level of sCD93 was elevated in patients with systemic sclerosis and correlated with disease severity (Yanaba et al. 2012), and it was also associated with the disease status of diabetic nephropathy and asthma (Sigari et al. 2016; Lee et al. 2020; Piani et al. 2023). Additionally, sCD93 could ameliorate inflammatory diseases by sequesters high-mobility group box 1 protein (HMGB1) (Huang et al. 2023). Although a recent study indicated that CD93 is important for maintaining endothelial barrier function and limiting metastatic dissemination (Vemuri et al. , 2024), a series of experimental research focusing on non-metastatic tumor found either knockout or blockade using a monoclonal antibody significantly reducing neovascular formation and tumor growth. Mechanistically, CD93 interacts with extracellular ligands, Insulin Like Growth Factor Binding Protein 7 (IGFBP7) and Multimerin-2 (MMRN2), and activates downstream signaling pathways to promote β1 integrin activation, actin reorganization, and further vascular maturation (Lugano et al. 2018; Li et al. 2023a). More importantly, CD93 blockade not only alleviates tumor growth and invasion but also shifts the tumor microenvironment toward immune infiltration and enhanced anti-tumor responses (Sun et al. 2021).

Accordingly, two bioinformatic studies have suggested the overexpression of CD93 in gastric adenocarcinoma, demonstrating strong associations between CD93 expression and tumor microenvironment (Li et al. 2023b; Wu et al. 2023). However, these findings were limited at the RNA level, and the clinical significance of CD93 in gastric adenocarcinoma has not been validated. To address this, we first assessed the expression and clinical correlation of CD93 in gastric adenocarcinoma using data from The Cancer Genome Atlas Program (TCGA). Next, we involved 404 patients with gastric adenocarcinoma to investigate the expression of CD93 at the protein level and evaluated its clinical pathological role.

## Materials and methods

### Analysis of the association between CD93 and clinicopathologic features in gastric adenocarcinoma

The gene expression and related clinicopathologic associations of CD93 in stomach adenocarcinoma (STAD) were analyzed using online tools from the University of Alabama at Birmingham (UALCAN, https://ualcan.path.uab.edu/analysis.html). This platform has integrated data from TCGA, encompassing gene expression converted to transcripts per million (TPM) and clinicopathologic variables, including disease stage, status of TP53 mutation, Helicobacter pylori infection, and overall survival (Chandrashekar et al. 2022, 2017).

### Patients and tissue samples

A total of 404 formalin-fixed paraffin-embedded gastric tissues were collected from patients undergoing radical gastrectomy for primary GC between January 2016 and December 2017 at the Department of General Surgery, Heping Hospital Affiliated to Changzhi Medical College. The inclusion criteria were (1) histological diagnosis of gastric adenocarcinoma, (2) harvesting of at least 15 lymph nodes, (3) absence of history of other tumors, and (4) availability of complete clinicopathological data and follow-up for a minimum of five years. Patients who received neoadjuvant radiotherapy, chemotherapy, or chemoradiotherapy were excluded because these treatments might lead to morphological changes and affect the overall prognosis. Tissues from tumor and tumor-adjacent regions (< 2 cm distance from tumor tissue) were collected and possessed at the same time. The baseline information for recruited patients was collected from hospital discharge records, clinical charts, and through face-to-face contact. The follow-up information was collected through outpatient visiting and remote contact. TNM-staging was performed according to the Union for International Cancer Control system 8th edition. Pathological information has been checked by two senior pathologists (YW and JW). This study has been approved by the Ethics Committee in Heping Hospital. All patients gave written informed consent before participation.

### Immunohistochemistry

The gastric adenocarcinoma tissue samples were fixed in 10% formalin and embedded in paraffin. The formalin-fixed paraffin-embedded tissues were continually cut into 3-μm sections for immunohistochemical staining. The slides were baked for 2 h at 65 °C, dewaxed, and rehydrated using xylene and graded alcohols. After antigen retrieval (pH = 6.0) and incubation with 3% H_2_O_2_, the tissues were blocked with 10% non-immune rabbit or goat serum for one hour at room temperature. Subsequently, the tissues were incubated with goat anti-human CD93 (1:100; AF2379, R&D Systems, Minneapolis, MN) or rabbit anti-human CD31 (1:500, 28,083–1-AP, Proteintech) at 4 °C overnight. Next, after washing with 1 × PBS three times, the tissues were incubated with horse radish peroxidase (HRP)-conjugated rabbit anti-goat antibody (GB23204, Servicebio, Wuhan, China) or HRP-conjugated goat anti-rabbit antibody (GB23303, Servicebio, Wuhan, China) at room temperature for one hour before staining with diaminobenzidine (DAB; G1211, Servicebio, Wuhan, China) and counterstaining with hematoxylin. After cleaning with tap water, the slides were dehydrated and placed in a gradient of alcohol and an environmentally friendly translucent liquid. The cover slides were mounted and examined under a microscope (E100; Olympus, Tokyo, Japan).

### Scoring of immunohistochemistry

**Fig. 1:**
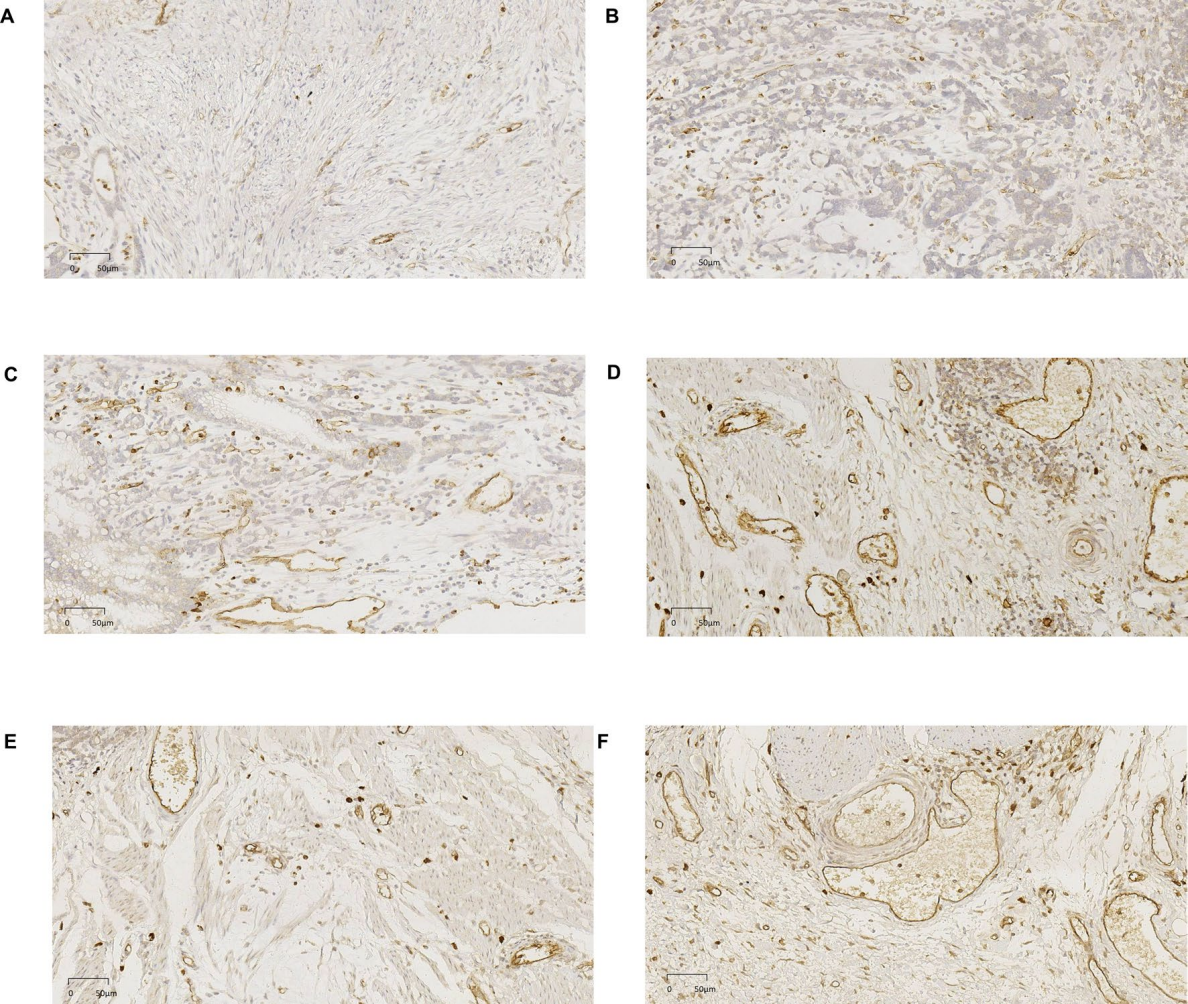
Representative images of immunohistochemistry score (**A**–**D**: staining intensity score 1–4, E–F: extent scoring 0–1)

Two experienced pathologists independently assessed the staining intensity and relative percentage of immunostained cells. To ensure a comprehensive evaluation, 10 consecutive representative high-magnification fields of view (× 400) were observed for each tissue section. These areas included regions with varying staining intensities and percentages of positive cells. By employing this rigorous evaluation process, we ensured the accuracy and reliability of CD93 expression assessment in gastric adenocarcinoma tissues (Bao et al. 2016). For extent scoring, the evaluation was standardized using the following percentage-based approach: 0, no CD93 staining; 1, 1–25% of cells were positive for CD93; 2, 26–50% of cells were positive; 3, 51–75% of cells were positive; and 4, 76–100% of cells were positive. The staining intensity was determined using a semi-quantitative approach, where the pathologists assigned a score of 2 for strong staining, 1 for weak staining, or 0 for negative staining (Fig. 1). The final expression score for each sample was derived by calculating the product of the intensity and the extent scores. This combined score considers both the staining intensity and the proportion of cells showing positive staining and provides a comprehensive representation of CD93 expression levels in gastric adenocarcinoma tissues. Using this scoring method, we aimed to obtain a quantitative and reliable assessment of CD93 expression that could contribute to a more accurate analysis of its potential role in gastric adenocarcinoma (You et al. 2015). Samples with a final score < 4 were categorized as CD93-low expression, whereas samples with a final score of 4–8 were categorized as high expression.

### Statistical methods

This study conducted all statistical analyses using GraphPad Prism 8.3 (GraphPad Software, San Diego, CA), EmpowerStats (X&Y solutions, Inc., Boston MA), and R 4.1.0 (R Foundation, Vienna, Austria). Depending on the specific comparison being made, between-group and within-group gene differences were assessed using either the Mann–Whitney test or the Kruskal–Wallis test. A Cox proportional hazards regression model was used to examine the association of CD93 with overall survival. Variables with P < 0.05 in the univariate analyses remained in the multivariate Cox proportional hazards regression model. Patient survival was analyzed using Kaplan–Meier curves, and the difference between survival was determined using the log-rank method. Each test with P < 0.05 was considered as statistically significant.

## Results

### CD93 expression and clinicopathologic significance in gastric adenocarcinoma

**Fig. 2:**
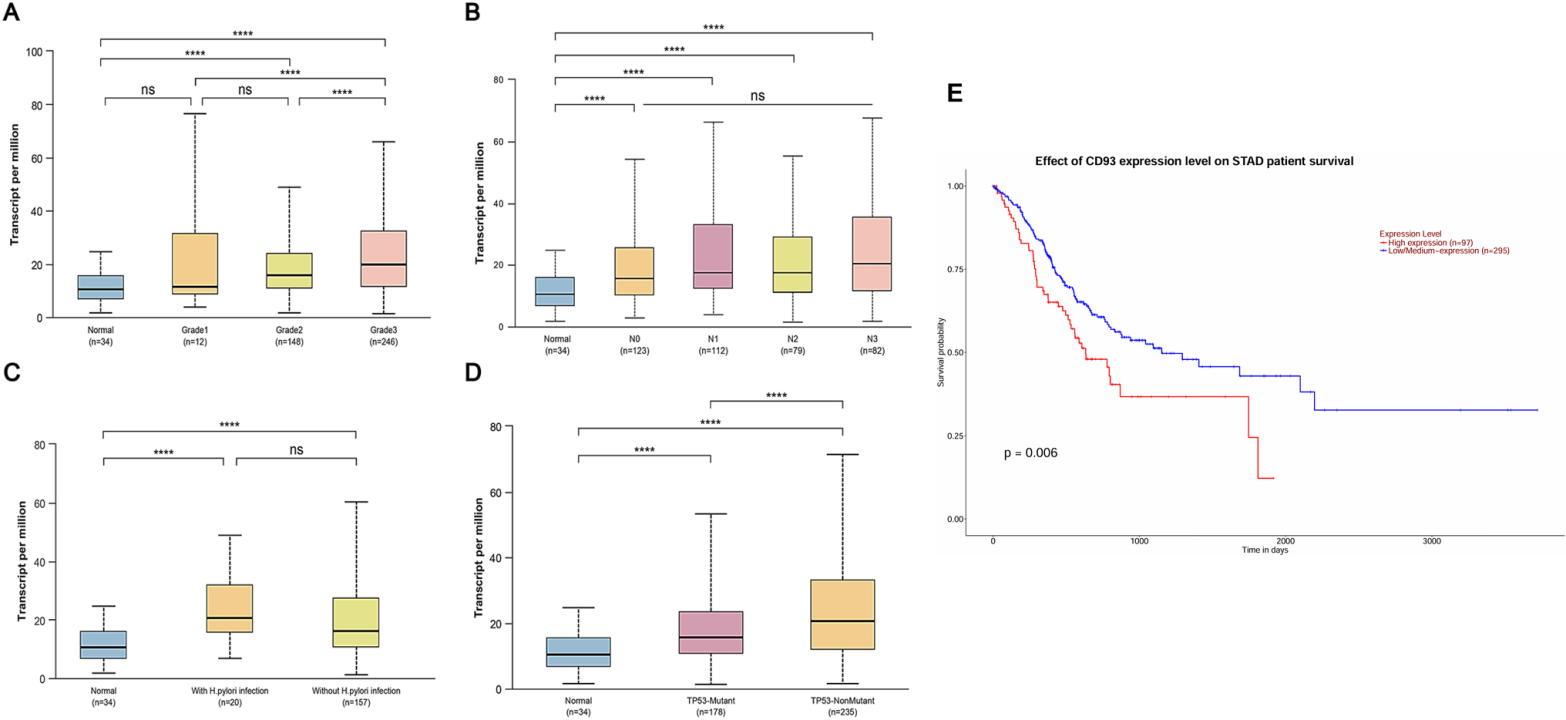
Gene expression and clinicopathological association of CD93 in stomach adenocarcinoma based on the Cancer Genome Atlas dataset. **A** The gene expression of CD93 in stomach adenocarcinoma across various tumor stages. **B** The gene expression of CD93 in stomach adenocarcinoma at different stages of lymph node metastasis. **C** Comparison of the gene expression of CD93 in stomach adenocarcinoma with and without Helicobacter pylori infection; **D** Comparison of the gene expression of CD93 in stomach adenocarcinoma with and without TP53 mutations; **E** Comparison of overall survival in patients with higher and lower gene expression of CD93 in stomach adenocarcinoma. P values for comparing gene expression of CD93 in different groups were calculated using Mann–Whitney *U* test. P value for comparing overall survival in different groups was calculated using log-rank test. *P < 0.05, **P < 0.01, ***P < 0.001, ****P < 0.0001, *ns* no statistically significant difference. P < 0.05 was considered significant

We first estimated the gene expression of CD93 in gastric adenocarcinoma using data from TCGA. As a result, we found that the gene expression of CD93 in gastric adenocarcinoma was significantly higher than that in normal tissues (Fig. 2A). Furthermore, after grouping STAD patients based on their tumor stage, we found that the gene expression of CD93 was comparable between normal tissues and stage one STAD tissues, which were significantly lower than that in STAD tissues with higher stages (range from stage two to four). However, CD93 gene expression within the tumor did not differ when the stage of STAD was over two (Fig. 2B). Next, we determined the potential clinical significance of CD93 by incorporating relevant clinicopathological data. Consequently, STAD patients with tumor grade three (T3) exhibited significantly higher expression of CD93 than did normal tissue and other groups. Interestingly, CD93 expression from STAD without TP53 mutation was higher than that with TP53 mutation (Fig. 2D). In contrast, the gene expression of CD93 was independent of the condition of lymph node metastasis or Helicobacter pylori infection (Fig. 2C). We further evaluated the prognostic role of CD93 for STAD (Fig. 2E). As a result, we observed a strong trend toward a poorer prognosis among patients with higher gene expression of CD93 (P = 0.006).

### CD93 expression was increased in gastric adenocarcinoma tissues

**Fig. 3:**
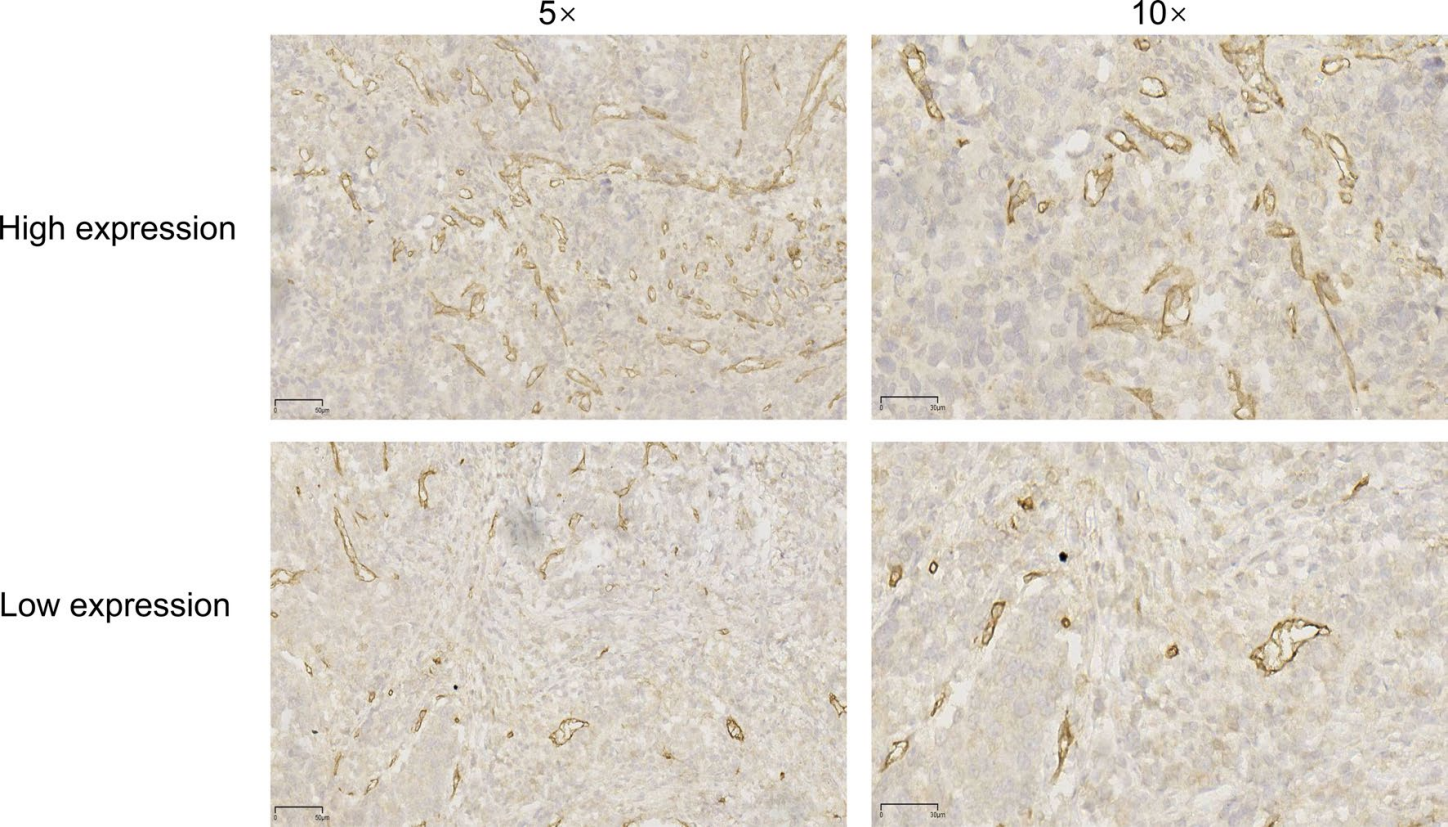
Representative immunohistochemical staining of different levels of CD93 expression in gastric adenocarcinoma

**Fig. 4:**
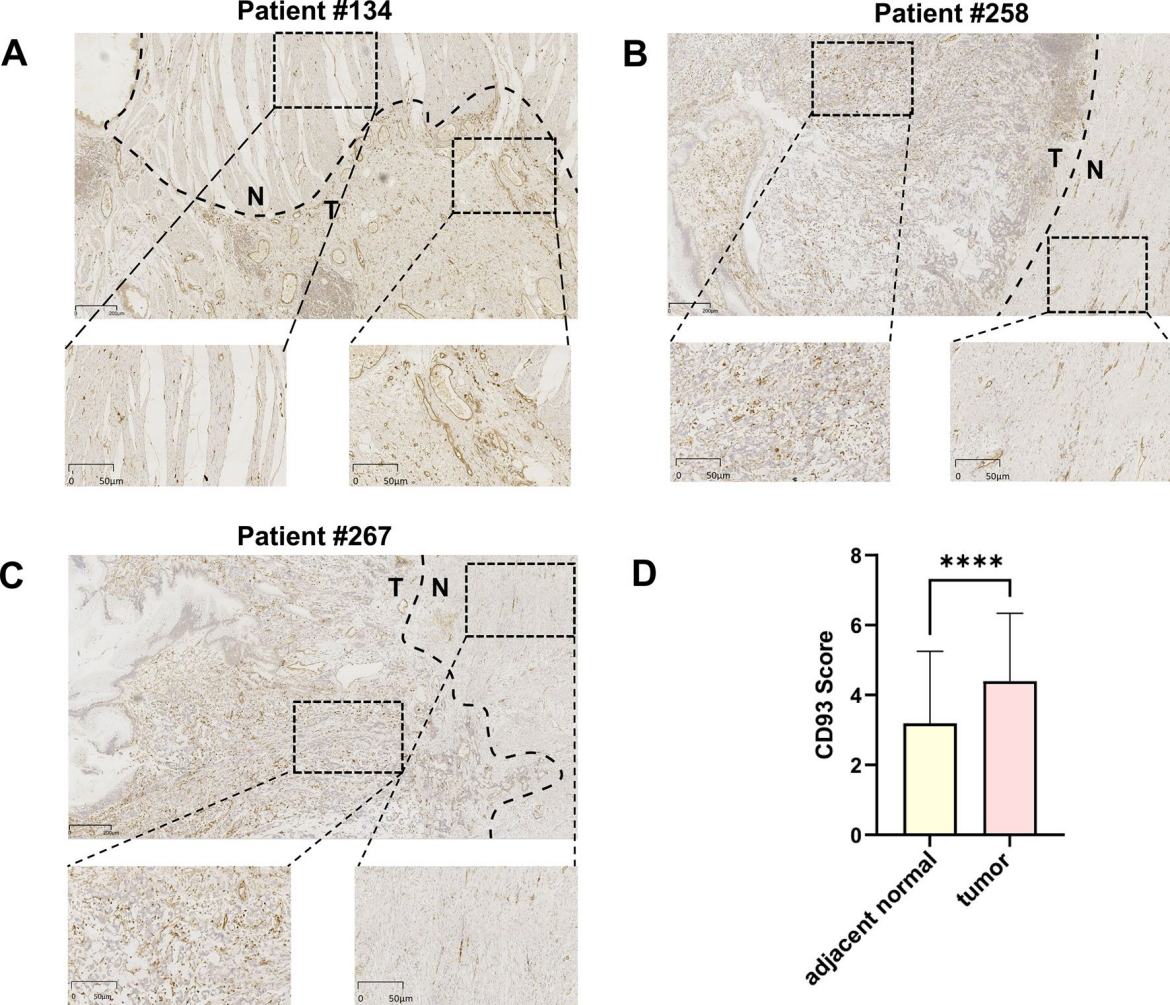
**A**–**C** Representative immunohistochemical staining of CD93 in gastric adenocarcinoma and adjacent normal gastric tissues. **D** Quantification and comparison of CD93 expression in gastric adenocarcinoma and adjacent normal gastric tissues. ****P < 0.0001, *T* tumor, *N* normal

**Fig. 5:**
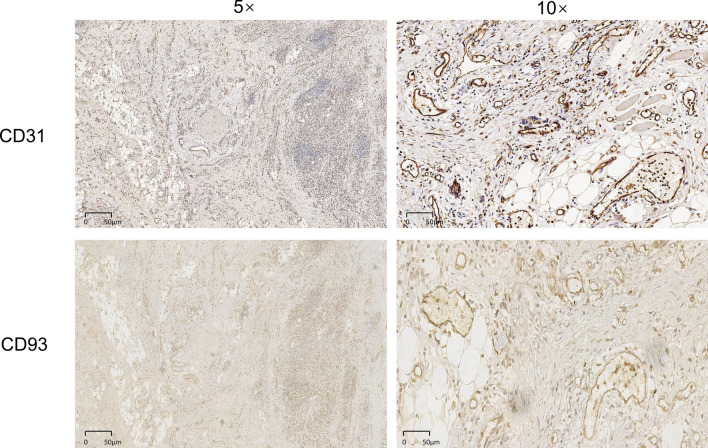
Representative immunohistochemical staining of CD31 **A**, **B** and CD93 **C**, **D** in gastric adenocarcinoma

To validate the findings from the public database, we further performed an immunohistochemistry assay to investigate the expression of CD93 in gastric adenocarcinoma. Notably, although CD93 expression was highly variable among these patients with gastric adenocarcinoma, we observed that the expression of CD93 in tumors was significantly higher than that in adjacent normal tissues (Fig. 3, Fig. 4). Furthermore, CD93 was mainly expressed on the membranes of vessel endothelial cells, which was consistent with the findings of previous studies. By contrast, only mild and rare expression of CD93 was found on some lymphocytes and tumor cells, respectively. To further confirm the dominant vascular expression of CD93, we performed immunohistochemistry assay of CD31 on the sequential-cut slides. As expected, we found that CD93 was mainly expressed on the membrane of CD31^+^ vascular endothelial cells (Fig. 5).

Taken together, our data showed that CD93 was overexpressed in patients with gastric adenocarcinoma and was mainly expressed on CD31^+^ vascular endothelial cells.

### Correlation between CD93 expression level and clinicopathologic features of gastric adenocarcinoma

**Fig. 6:**
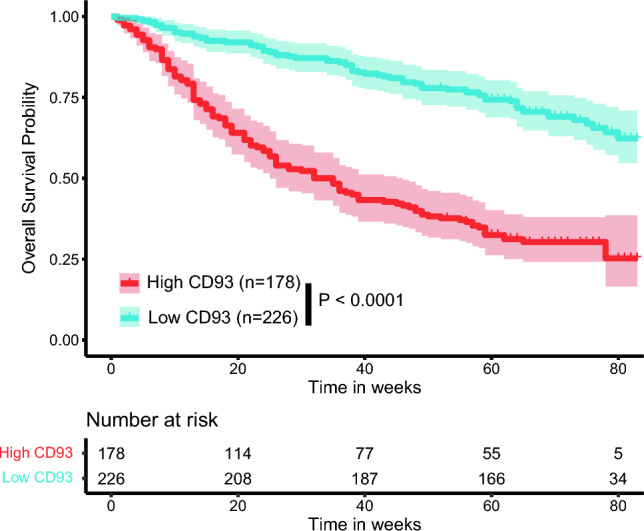
Comparison of overall survival in patients with higher and lower expression of CD93 in gastric adenocarcinoma

Furthermore, we investigated the potential clinical significance of CD93 by incorporating relevant clinicopathological information. High expression of CD93 was observed in 43.1% (n = 174) of the whole population (Table 1). Notably, we found that patients with higher expression of CD93 demonstrated significantly lower overall survival than did those with lower expression (33.37 ± 24.80 months vs. 63.24 ± 17.06 months, P < 0.001) (Fig. 6). Additionally, gastric adenocarcinoma patients with higher expression of CD93 demonstrated deeper invasion, higher proportion of lymph node metastasis, higher possibility of developing tumor thrombus, resulting in advanced tumor stage.

**Table 1:** Comparison of clinicopathological characteristics in gastric adenocarcinoma patients stratified by CD93 expression

Clinicopathological feature	Number (Percentage of total)	CD93 low expression N = 230	CD93 high expression N = 174	*P*-value
Gender, %				0.360
Male	313 (77.48%)	185 (81.86%)	128 (71.91%)	
Female	91 (22.52%)	41 (18.14%)	50 (28.09%)	
Age(years), %				0.713
< 60	131 (32.43%)	75 (33.19%)	56 (31.46%)	
≥ 60	273 (67.57%)	151 (66.81%)	122 (68.54%)	
Tumor location, %				0.106
Esophagogastric junction	245 (60.64%)	145 (64.16%)	100 (56.18%)	
Corpuscles	79 (19.55%)	36 (15.93%)	43 (24.16%)	
Gastric sinus	80 (19.8%)	45 (19.91%)	35 (19.66%)	
Stereotyping, %				0.924
Ulcerated	322 (79.70%)	182 (80.53%)	142 (79.78%)	
Mycosis fungoides	24 (5.94%)	12 (5.31%)	12 (6.74%)	
Infiltration	29 (7.18%)	16 (7.08%)	13 (7.30%)	
Vesicular	100 (24.75%)	16 (7.08%)	11 (6.18%)	
Histological differentiation, %				0.752
Low	3 (0.74%)	2 (0.88%)	1 (0.56%)	
Moderate	159 (39.36%)	92 (40.71%)	67 (37.64%)	
High	242 (59.90%)	132 (58.41%)	110 (61.80%)	
Tumor diameter (cm), %				0.678
< 5	74 (18.32%)	43 (19.03%)	31 (17.42%)	
≥ 5	330 (81.68%)	183 (80.97%)	147 (82.58%)	
Infiltration depth, %				0.714
T1 + T2	92 (22.77%)	53 (23.45%)	39 (21.91%)	
T3 + T4	312 (77.23%)	173 (76.55%)	139 (78.09%)	
Lymphatic metastasis, %				**0.018**
No	167 (41.34%)	105 (46.46%)	62 (34.83%)	
Yes	237 (58.66%)	121 (53.54%)	116 (65.17%)	
Distant metastasis, %				0.431
No	403 (99.75%)	226 (100.00%)	177 (99.44%)	
Yes	1 (0.25%)	0 (0.00%)	1 (0.56%)	
TNM stages, %				**0.002**
I + II	185 (45.79%)	119 (52.65%)	66 (37.08%)	
III + IV	219 (54.21%)	107 (47.35%)	112 (62.92%)	
Intravascular tumor thrombus, %				**0.030**
No	370 (91.58%)	213 (94.25%)	157 (88.20%)	
Yes	34 (8.42%)	13 (5.75%)	21 (11.80%)	
Neurological violation, %				**0.041**
No	331 (81.93%)	193 (85.40%)	138 (77.53%)	
Yes	73 (18.07%)	33 (14.60%)	40 (22.47%)	
Harmful eating habit, %				**0.012**
No	143 (35.4%)	68 (30.09%)	75 (42.13%)	
Yes	261 (64.6%)	158 (69.91%)	103 (57.87%)	
HP/History of gastric ulcers, %				**0.011**
No	205 (50.74%)	102 (45.13%)	103 (57.87%)	
Yes	199 (49.26%)	124 (54.87%)	75 (42.13%)	
Survival time (months), %	50.38 ± 25.47	60.48 ± 20.63	37.55 ± 25.31	** < 0.001**
CEA (ug/L), %	10.62 ± 41.71	11.73 ± 45.55	9.21 ± 36.32	0.547
CA-199 (ug/L), %	35.34 ± 106.39	35.00 ± 109.76	35.76 ± 102.27	0.943
CA-724 (ug/L), %	4.99 ± 25.01	4.68 ± 21.43	5.38 ± 28.99	0.781

*P* < 0.05 was shown in bold

### CD93 expression is an independent predictor of the prognosis in stomach adenocarcinoma

We finally performed Cox regression to determine whether CD93 expression was a predictor of the prognosis of patients with gastric adenocarcinoma. Among all clinicopathological variables evaluated, univariate Cox regression showed that presence of lymph node metastasis (HR = 1.72, P < 0.001), higher depth of tumor invasion (HR = 1.52, P = 0.022), presence of distant metastasis (HR = 14.12, P = 0.009), higher tumor grade (HR = 2.01, P < 0.001), presence of intravascular tumor thrombus (HR = 1.80, P = 0.011), neuron invasion (HR = 1.45, P = 0.033), as well as higher CD93 expression (HR = 3.53, P < 0.001), were significantly associated with poorer overall survival. Further multivariate Cox regression revealed that only higher tumor grades (HR = 1.77, P < 0.001) and higher CD93 expression (HR = 3.26, P < 0.001) were independent variables that significantly associated with overall survival (Table 2).

**Table 2:** Univariate and multivariate Cox analysis for overall survival in all patients with gastric adenocarcinoma

	Univariate analysis		Multivariate analysis	
Factor	HR	*P* value	HR	*P* value
Gender				
Male	1 [Reference]	0.77		
Female	1.05 (0.75–1.46)			
Age(years)				
< 60	1 [Reference]	0.66		
≥ 60	1.07 (0.79–1.45)			
Tumor location				
Esophagogastric junction	1 [Reference]			
Corpuscles	1.49 (1.05–2.11)	**0.024**	1.27 (0.89–1.83)	0.19
Gastric sinus	1.19 (0.83–1.70)	0.34	1.20 (0.83–1.73)	0.33
Stereotyping				
Ulcerated	1 [Reference]		1 [Reference]	
Mycosis fungoides	1.39 (0.82–2.36)	0.22	1.31 (0.77–2.23)	0.32
Infiltration	1.14 (0.67–1.94)	0.62	1.22 (0.70–2.14)	0.48
Vesicular	0.38 (0.17–0.86)	**0.02**	0.46 (0.20–1.08)	0.09
Histological differentiation				
Low	1 [Reference]			
Moderate	1.20 (17–8.66)	0.85		
High	1.62 (0.23–11.59)	0.63		
Tumor diameter (cm)				
< 5	1 [Reference]	0.07		
≥ 5	1.44 (0.97–2.14)			
Infiltration depth				
T1 + T2	1 [Reference]	**0.022**	1 [Reference]	0.28
T3 + T4	1.52 (1.06–2.16)		0.77 (0.47–1.24)	
Lymphatic metastasis				
No	1 [Reference]	** < 0.001**	1 [Reference]	
Yes	1.72 (1.28–2.33)		0.54 (0.04–6.85)	0.63
Distant metastasis				
No	1 [Reference]	**0.009**	1 [Reference]	0.25
Yes	14.12 (1.92–103.66)		3.93 (0.42–29.14)	
TNM stages				
I + II	1 [Reference]	** < 0.001**	1 [Reference]	** < 0.001**
III + IV	2.01 (1.49–2.70)		1.77 (1.30–2.40)	
Intravascular tumor thrombus				
No	1 [Reference]	**0.011**	1 [Reference]	0.49
Yes	1.80 (1.14–2.83)		1.24 (0.68–2.27)	
Neuron invasion				
No	1 [Reference]	**0.033**	1 [Reference]	0.80
Yes	1.45 (1.03–2.05)		0.94 (0.59–1.49)	
Harmful eating habit				
No	1 [Reference]	**0.018**	1 [Reference]	0.57
Yes	0.71 (0.53–0.94)		0.92 (0.67–1.24)	
HP/History of gastric ulcers				
No	1 [Reference]	0.08		
Yes	0.78 (0.59–1.03)			
CD93 expression				
Low	1 [Reference]	** < 0.001**	1 [Reference]	** < 0.001**
High	3.53 (2.63–4.73)		3.26 (2.32–4.39)	

*P* < 0.05 was shown in bold

## Discussion

In this study, we observed significantly higher expression of CD93 in gastric adenocarcinoma when compared to that of adjacent normal tissues, with CD93 primarily expressed on the membrane of vascular endothelial cells. Furthermore, elevated CD93 expression was correlated with more severe manifestations of tumor progression and strongly associated with poorer overall survival. More importantly, multivariate analysis underscored CD93 expression as an independent prognostic predictor for gastric adenocarcinoma patients.

The carcinogenesis of GC was typically characterized by dysregulated transcriptional and epigenetic programs, as well as a shift in the metabolic process towards aerobic glycolysis, by which tumor cells could fuel the rapid metabolic demands for malignant cell proliferation. This process was commonly coupled with disorganized vasculature, which hindered the delivery of oxygen and nutrients. To address this, malignant cells orchestrated neovascular formation to uptake required nutrients (Yuan et al. 2022). However, due to dysregulated levels of proangiogenic and antiangiogenic factors, the intratumor neovascular architecture exhibited both structural and functional abnormalities. Specifically, tumor blood vessels displayed morphologically tortuous and unevenly distributed, leading to impaired perfusion capacity. Moreover, they expressed lower levels of cell adhesion molecules, which limited immune cell infiltration and further restricted the efficiency of immunotherapy. Consequently, tumor vasculature further helped malignant cells to create a hostile tumor microenvironment that promotes impaired anti-tumor immunity.

Although the impact of angiogenesis in the development of gastric adenocarcinoma has been widely acknowledged, the critical driving factors of angiogenesis remain to be elucidated. Notably, the VEGF family, mainly derived from tumor cells, was thought to be the most important driver of neovascularization in GC. Moreover, it has been found that HP infection directly induced the production of VEGF in tumor cells (Liu et al. 2016). Furthermore, early studies suggested that advanced GCs with positive staining of VEGF were larger, more invasive, and had lower survival rates than did VEGF-negative ones (Kakeji et al. 2002; Lian et al. 2019; Kosaka et al. 2007). In addition to tumor cells, nonparenchymal cells within the tumor also demonstrated profound effects on the angiogenesis of GC. For instance, tumor-infiltrating neutrophils and macrophages were found to promote neovascular formation by releasing a series of cytokines, including interleukin-17 and transforming growth factor β1 (Li et al. 2017; Qiu et al. 2022). Recent studies also highlighted the critical role of cancer-associated fibroblasts in driving tumor angiogenesis by secreting chemokines and VEGFA (Ma et al. 2023; Chen et al. 2021).

Therefore, VEGF blockade, encompassing monoclonal antibody-based agents and VEGF receptor tyrosine kinase inhibitors, is currently the primary strategy for vascular normalization in GC (Yu et al. 2016). To some extent, these treatments have alleviated the progression of GC progression, but their effect on long-term prognosis did not show satisfactory outcomes. Specifically, adding bevacizumab to chemotherapy demonstrated benefit for improving progression-free survival, and monotherapy with Ramucirumab could prolong both overall and progression-free survival for patients with advanced GC, implying (Ohtsu et al. 2011; Fuchs et al. 2014). Furthermore, the combination of Ramucirumab with paclitaxel significantly increased overall survival and has been recommended as second-line therapy for GC (Wilke et al. 2014; Xu et al. 2021). However, neither cisplatin plus fluoropyrimidine chemotherapy nor front-line mFOLFOX6 improved survival when combined with Ramucirumab (Fuchs et al. 2019; Yoon et al. 2016). More importantly, all of the abovementioned treatments failed to improve the median survival over six months. Therefore, novel targets for vascular normalization were required for GC treatment. Recent experimental studies have revealed that several peptides demonstrated promising performance in suppressing GC progression and metastasis by inhibiting angiogenesis, including Activin A, TCP-1, and JP3, but their application might be limited by challenges related to precise delivery and loss-of-target (Kaneda et al. 2011; Lu et al. 2017; Chen et al. 2020). In this regard, we focused on potential molecules that were highly expressed on the membrane of intratumor vascular endothelial cells but not in vital tissues.

CD93, one of the top genes representing a common angiogenic signature in human tumors, was found to promote angiogenesis in both inflammatory and malignant diseases (Masiero et al. 2013). CD93 exhibited relatively low expression on vascular endothelial cells under physiological conditions, providing a basis for distinguishing pathogenic vessels from normal ones (Khan et al. 2019). Mechanistically, upon activation by extracellular ligands IGFBP7 and MMRN2, CD93 enhanced integrin signaling and phosphorylation of focal adhesion kinase in endothelial cells, further promoting fibronectin fibrillogenesis. Additionally, CD93 bound to downstream adaptor proteins Cbl/Crk, regulating cell polarity and migration (Lugano et al. 2018; Barbera et al. 2021; Xu et al. 2023). More importantly, the blockade of CD93 significantly normalized intratumoral vascular structure and promoted the efficiency of drug delivery and immunotherapy (Sun et al. 2021). Accordingly, using both the public RNA-seq dataset and our observations from immunohistochemistry, we determined that the expression of CD93 was significantly upregulated in gastric adenocarcinoma tissues and was mainly expressed on the membrane of CD31^+^ vascular endothelial cells. Furthermore, we not only characterized a more severe clinical phenotype in patients with higher CD93 expression but also identified CD93 as an independent predictor of the prognosis in patients with gastric adenocarcinoma.

Nevertheless, this study has several limitations that should be acknowledged. First, given the retrospective design, the clinical significance of CD93 should be further tested in prospective cohorts incorporating diverse populations. Second, since all samples were collected after surgery, the expression of CD93 has not been determined in gastric biopsy tissues of premalignant lesions, such as gastritis and intestinal metaplasia. Third, further enrollment of participants who have received immunotherapy is necessary to investigate the potential impact of CD93 on immunotherapy response.

In conclusion, our study indicated a significantly higher expression of CD93 in gastric adenocarcinoma and found that the level of CD93 expression was strongly associated with clinical severity and prognosis.

## Data Availability

Data are available on reasonable request.
